# Modulation of the maternal immune system by the pre-implantation embryo

**DOI:** 10.1186/1471-2164-11-474

**Published:** 2010-08-13

**Authors:** Caroline G Walker, Susanne Meier, Mathew D Littlejohn, Klaus Lehnert, John R Roche, Murray D Mitchell

**Affiliations:** 1 DairyNZ Ltd., Hamilton, New Zealand; 2 Liggins Institute, The University of Auckland, Grafton, New Zealand; 3 ViaLactia Biosciences, Auckland, New Zealand; 4 UQ Centre for Clinical Research, The University of Queensland, Brisbane, Queensland

## Abstract

**Background:**

A large proportion of pregnancy losses occur during the pre-implantation period, when the developing embryo is elongating rapidly and signalling its presence to the maternal system. The molecular mechanisms that prevent luteolysis and support embryo survival within the maternal environment are not well understood. To gain a more complete picture of these molecular events, genome-wide transcriptional profiles of reproductive day 17 endometrial tissue were determined in pregnant and cyclic Holstein-Friesian dairy cattle.

**Results:**

Microarray analyses revealed 1,839 and 1,189 differentially expressed transcripts between pregnant and cyclic animals (with ≥ 1.5 fold change in expression; P-value < 0.05, MTC Benjamini-Hochberg) in caruncular and intercaruncular endometrium respectively. Gene ontology and biological pathway analysis of differentially expressed genes revealed enrichment for genes involved in interferon signalling and modulation of the immune response in pregnant animals.

**Conclusion:**

The maternal immune system actively surveys the uterine environment during early pregnancy. The embryo modulates this response inducing the expression of endometrial molecules that suppress the immune response and promote maternal tolerance to the embryo. During this period of local immune suppression, genes of the innate immune response (in particular, antimicrobial genes) may function to protect the uterus against infection.

## Background

Over the past three decades, there has been a coincidental decline in fertility associated with genetic selection for increased milk production. It is estimated that approximately 50% of the potential profitability from genetic selection for milk production is lost due to a reduction in fertility [1].

The fertilisation rate for lactating dairy cattle is around 90% and does not differ between low-moderate and high-producing animals when managed under pastoral conditions[2]. However, the calving rate in lower producing animals is approximately 55%, whereas for high-producing animals, this rate is approximately 35%[2]. Pregnancy losses are thought to occur primarily during the pregnancy recognition/pre-implantation period [2], making studies of endometrial gene expression critical to further understanding of pregnancy establishment, recognition and maintenance within the bovine reproductive cycle.

Successful pregnancy in mammals requires both a viable embryo and a receptive endometrium. Synchronous signalling between the endometrium and embryo during the pre-implantation period is critical for normal embryo development, implantation of the embryo, and placentation [3]. The early embryo is nourished by secretions (histotroph) from the uterine glands (intercaruncular endometrium) and during implantation forms a close physical association (attachment) with the caruncular endometrium [4]. Pregnancy thus represents an immunological contradiction, in that the immunologically foreign embryo is able to form a close physical relationship with the maternal endometrium that lasts throughout pregnancy. Under normal circumstances, a foreign tissue would likely be rejected by the recipient unless the immune system was significantly suppressed or tolerant to the tissue. That the embryo can survive in the presence of the maternal immune system has lead to the hypothesis that the uterus is an immunologically privileged site [5].

Several mechanisms have been proposed to account for the ability of the embryo to survive in the maternal environment including: antigenic immaturity of the conceptus (the bovine trophoblast, like other mammalian species, does not express classical polymorphic major histocompatibility complex (MHC) class 1 proteins in areas in contact with the maternal endometrium during early pregnancy [5]) and maternal immunological inertness to the conceptus or localised immune tolerance [6].

Local immunosuppression, required for establishment and maintenance of pregnancy, would leave the uterus vulnerable to infection. An increase in innate immune activity may be expected to protect the uterus from infection.

The aim of this study was to identify molecules and pathways involved in pregnancy recognition and maintenance and, in particular to characterise the local immune response that occurs in the endometrium as a consequence of pregnancy. The transcriptional response to the presence of an embryo was characterised during the pre-implantation period in dairy cows. Novel molecules and pathways potentially involved in mechanisms the embryo uses to evade the maternal immune system were identified.

## Results

### Differentially expressed genes

**Figure 1:**
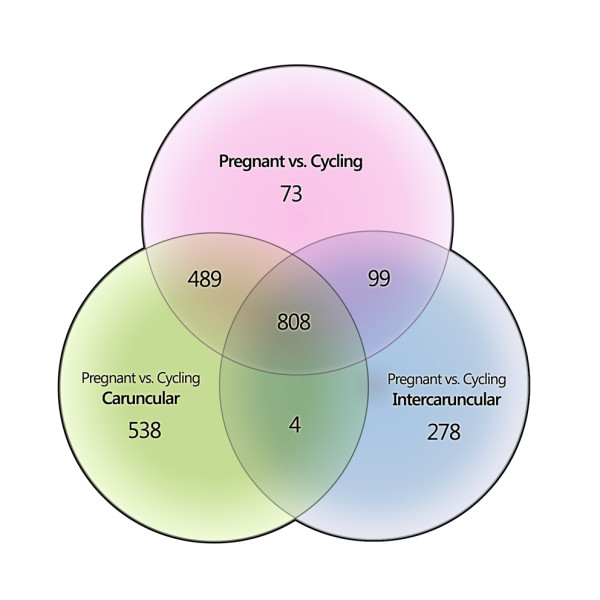
**Number of genes differentially expressed between pregnant and cyclic animals in both and individual tissue types**. Genes with ≥ 1.5 fold change in expression; P-value < 0.05, Benjamini-Hochberg MTC.

Microarray analyses revealed 1,839 and 1,189 differentially expressed transcripts between pregnant and cyclic animals (with ≥ 1.5 fold change in expression; P-value < 0.05, MTC Benjamini-Hochberg) in caruncular and intercaruncular endometrium respectively (Additional file 1, Table S1). The majority of transcripts were up-regulated in pregnant animals (1027 in caruncular, 633 in intercaruncular). Some genes were differentially expressed between pregnant and cyclic animals in either the caruncular or intercaruncular tissues. Of these, 1027 (480 up-regulated in pregnant) differentially expressed transcripts were identified in caruncular endometrium only, and a further 377 (86 up-regulated in pregnant) differentially expressed transcripts were identified only in intercaruncular tissue (Figure 1).

### Molecular and biological function and pathway analysis

**Figure 2:**
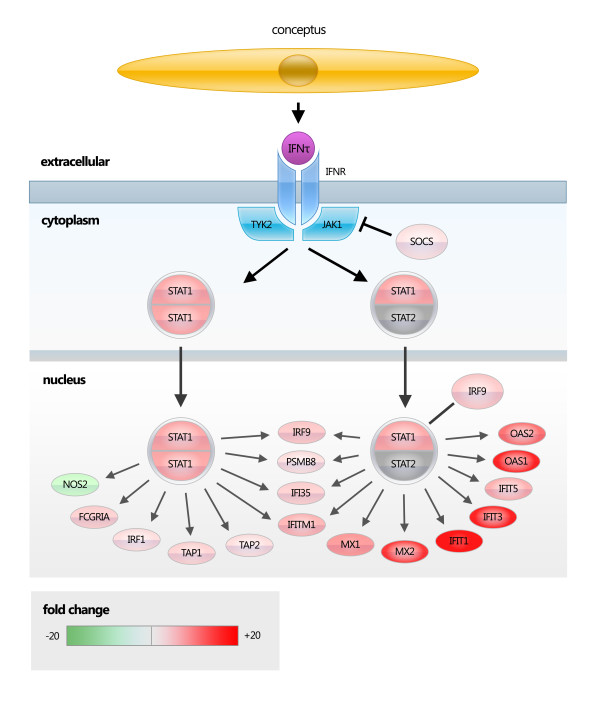
**Interferon signalling cascade**. Genes differentially expressed between pregnant and cyclic dairy cows in the study reported are shaded red (up-regulated in pregnant) and green (down-regulated in pregnant). The bovine embryo produces IFNτ which binds to type-1 interferon receptors, leading to the activation of the JAK-STAT (Janus kinase-signal transducer and activator of transcription) pathway and the synthesis of a range of interferon stimulated gene products.

**Figure 3:**
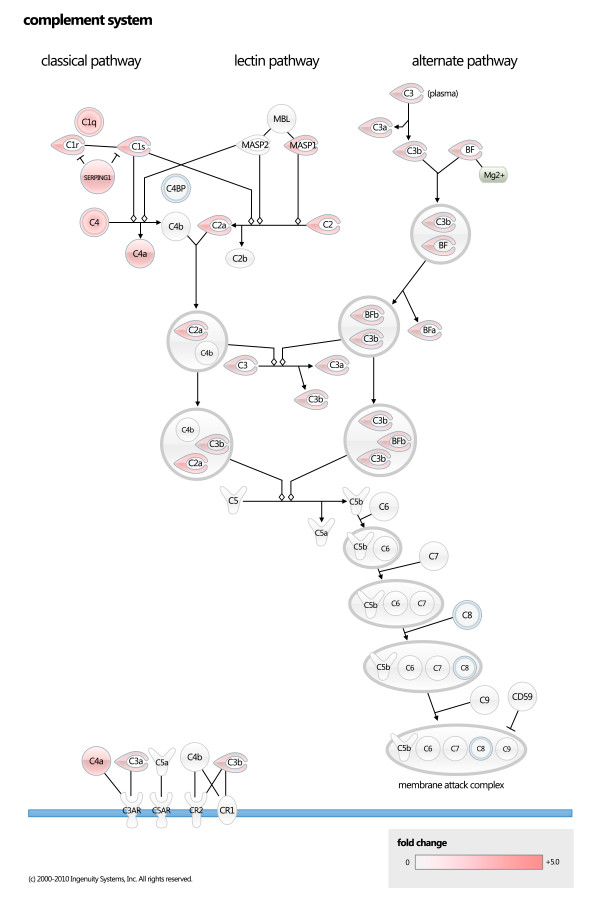
**Complement pathway**. Genes up-regulated in pregnant dairy cows are shaded red. The complement system is part of the innate immune response and can be activated in three ways: classical pathway, alternative pathway and lectin pathway, all of which converge at the level of C3 convertase. The Classical pathway begins with activation of the C1 complex (6xC1q, 2xC1r and 2xC1s) through the binding of C1q to antigen bound antibodies (IgG or IgM) or directly to the surface of a pathogen. Binding causes a conformational change in C1q which leads to activation of C1r and C1s (serine proteases), this leads to cleavage of C4 and C2. The cleavage products of C4 and C2 form C3 convertase cleaves C3, leading to the formation of C5 convertase that cleaves C5 and results in formation of the membrane attack complex[73].

Ingenuity pathway analysis (IPA) revealed molecular networks and pathways associated with genes that were differentially expressed in pregnant versus cyclic animals. 1,499 individual genes were contained within the Ingenuity database and were used for analysis. The four most statistically significant canonical pathways identified by IPA were interferon signalling (Figure 2), complement system (Figure 3), Role of pattern recognition receptors in the recognition of bacteria and viruses, and antigen presentation (Additional file 2, Table S2). The networks generated by IPA that contained the most significant number of genes with direct relationships (molecules that directly affect one another) were:

- **Network 1 **- Cell death, hematological disease, immunological disease (score = 49, 28 molecules)

- **Network 2 **- Infection mechanism, antimicrobial response, cell signaling (score = 45, 26 molecules)

- **Network 3 **- Infectious disease, cell morphology, cellular assembly and organization (score = 43, 26 molecules)

- **Network 4 **- Cellular growth and proliferation, connective tissue development and function, cell cycle (score = 23, 16 molecules)

- **Network 5 **- Lipid metabolism, molecular transport, small molecule biochemistry (score = 21, 15 molecules).

'Functional groups' and biological processes associated with pregnancy and estrous cycle progression (cycling animals) in the dairy cow during the preimplantation period were identified. Genes were assigned to molecular and biological functions using the PANTHER (Protein Analysis Through Evolutionary Relationships) classification system (http://www.pantherdb.org/). The biological process "immunity and defence" contained the most genes for this analysis. (Additional file 3, Table S3).

### Relative quantitative real time PCR confirmation of microarray results

**Figure 4:**
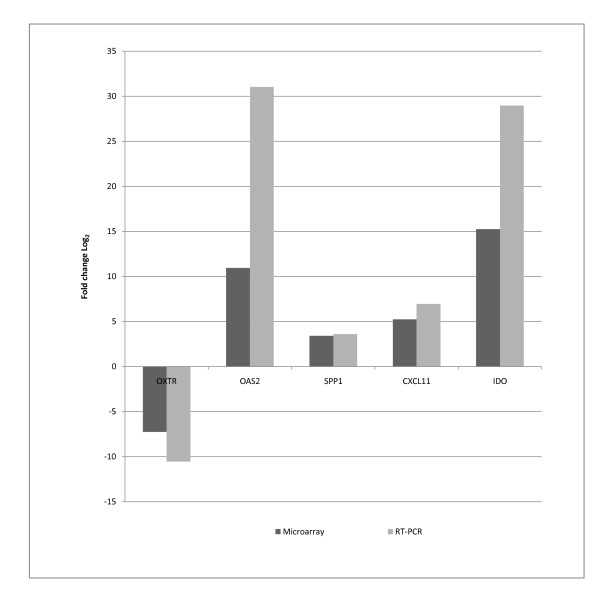
**RT-PCR confirmation of microarray**.

Relative qRT-PCR was used to confirm microarray results. 5 genes of interest (OXTR, IDO, SPP1, OAS2, and CXCL11) that were differentially expressed according to microarray analysis were quantified, and the correlation between qRT-PCR and microarray (Pearson correlation coefficient) calculated exceeded 0.75 for all genes tested (Table 1, Figure 4).

**Table 1:** RT-PCR confirmation of microarray

Fold change Pregnant vs Cyclic				Primer Sequences		
	**Microarray**	**RT-PCR**	**Correlation**	**Forward**	**Reverse**	**Probe**

OXTR	-7.26	-10.55	0.90	cgtgcagatgtggagtgtct	ttgcagcagctgttgagg	UPL#162

OAS2	10.94	31.05	0.93	tggacggtcaactacagttttg	ctgggtccaagatcacagg	UPL#69

SPP1	3.41	3.60	0.75	aagttccgccgatctaacg	cctcactctctatgtgtgatgtgaa	UPL#82

CXCL11	5.23	6.95	0.83	tgctgcaattgttcaaggtt	tctgccactttgactgctttt	UPL#81

IDO	15.25	28.97	0.76	acgtaggctttgctcttcca	gagatccaggcatcataagga	UPL#65

## Discussion

### Genes up-regulated in pregnant animals

In this study, endometrial expression profiles of day 17 pregnant and cyclic dairy cows were characterized and several genes and pathways that were differentially expressed between the two states were identified, providing insight into the molecular mechanisms active during this time. Genes and pathways involved in the maternal immune response to the presence of the embryo appear to be particularly important in early pregnancy, as these were some of the most up-regulated genes in pregnant animals. The immune response to pregnancy may be one of the key regulators of pregnancy maintenance, and deregulation of the immune response may be responsible, at least in part, for the large number of pregnancy losses that occur during this time. Both innate and adaptive immune system genes were differentially expressed during early pregnancy. Many genes of the adaptive immune response that were up-regulated may function to induce immune tolerance to the embryo, while genes of the innate immune response (in particular, antimicrobial genes) may function to protect the uterus against infection during a time of local immune suppression.

### Immune response and interferon signalling

Interferon stimulated genes (ISG) were among the most up-regulated group of genes in pregnant animals; this is consistent with maximal production of the pregnancy recognition signal interferon tau (IFNτ) by the embryo during this time. The most statistically significant networks up-regulated in pregnant animals were involved in the immune response, and immunity and defence were identified as the most abundant gene ontology terms.

Several ISGs were identified as being differentially expressed in pregnant and cyclic animals, in agreement with previous studies [7-11]. The current study, however, has identified several additional ISG not previously identified in the bovine endometrium (Figure 2, Additional file 2, Table S2). Many of these genes may function to provide localized immune system suppression to allow the embryo to survive within the uterus. For example, IFITM1 has recently been demonstrated to act as an immune suppressive molecule in gastric cancer cells [12]. The upregulation of IFITM1 in cancer cells was associated with an increase in migration and invasive capacity, and its action in gastric tumour cells was linked to suppression of NK cells [12]. Peptide Transporter 1 (TAP-1) and TAP-2 were up-regulated in pregnant animals in agreement with another study [13] along with MHC I A, and MHC I G. Upregulation of TAP proteins may be involved in local immune suppression, as upregulation of TAP1 has been associated with the capacity of natural killer cells to be non-cytotoxic [14]. Kalkunte et al 2009 demonstrated that the non-cytotoxic phenotype of uNK cells is achieved through the expression of vascular endothelial growth factor-C (VEGF-C), which enhances the resistance of trophoblast and endothelial cells to lysis through induction of the TAP-1 protein [14]. They reported that VEGF-C protected target endothelial and trophoblast cells from cytotoxic NK cells directly through induction of TAP-1.

Additionally, in agreement with a local immune suppression environment, IFIT1, OAS1 and OAS2 are up-regulated in the autoimmune disease systemic lupus [15]. OAS upregulation during early pregnancy is also involved in regulating the production of osteopontin (SPP1) [16,17], that was also up-regulated in pregnant animals. Upregulation of SPP1 in pregnant animals may have several functions, including promoting adhesion of the trophoblast to the endometrium, stimulating morphological changes in the trophoblast [18], and regulating the immune response. SPP1 is expressed and secreted by immune cells including T-lymphocytes, monocytes, macrophages and NK cells. SPP1 is presumed to regulate the TH1/TH2 balance and apoptosis[18]. SPP1 polymorphisms are associated with many immune-mediated inflammatory diseases [19] consistent with a local immune suppressive state. Quantitative trait (QTL) analyses in pigs have identified SPP1 as a candidate gene for reproductive performance [20].

Alternatively, upregulation of these genes may be an important mechanism to enhance the response to potential viral pathogens that the uterus may encounter during the time of local immune suppression that occurs in response to the embryo. This Hypothesis is supported by the upregulation of MX1 and MX2, both of which are up-regulated in response to viral infection [21,22]. MX2 expression is also up-regulated in peripheral blood leukocytes during pregnancy [23], suggesting the innate immune system is active during early pregnancy.

Several of the above genes are also thought to be important regulators of luteolysis [21]. For example, OAS inhibits prostaglandin F2α synthesis, possibly through alteration of arachidonic acid metabolism [24]. Interestingly, MX2 expression is lower in the uterus of pregnancies with cloned embryos than in embryos produced by IVF.

#### Cytokines, chemokines, and growth factors

Pregnancy requires a delicate balance between pro-inflammatory and anti-inflammatory molecules to maintain maternal immune system integrity, while preventing rejection of the embryo. Expression patterns of chemokines and their receptors during the implantation period suggest that they are involved in the regulation of embryo attachment as well as having immunomodulatory properties. Chemokines attract leukocytes to sites of inflammation, and contribute to their local activation.

Several chemokines were up-regulated in pregnant animals in agreement with other studies [25-28]. The CXCR3 ligands CXCL9, CXCL10 and CXCL11 were all up-regulated in pregnant animals, a difference that is more pronounced in the caruncular compared with intercaruncular tissue. CXCR3 is preferentially expressed on TH1 cells, and the expression of ligands for this receptor suggests there may be an influx of TH1 cells into the uterus. Upregulation of the CXCR3 receptor ligands and the influx of TH1 cells have been associated with allograft rejection [25,29]. However since CXCR3 was not up-regulated, it suggests that either TH1 cell numbers were not increased in the uterus of pregnant animals, or that they were not expressing this receptor. CXCR3 is also expressed in human uterine natural killer cells [30], so upregulation of these chemokines may function to attract the trophoblast and/or uNK cells. Several chemokines that were up-regulated in pregnant animals are known to attract immune tolerance promoting leukocytes, including TH2 and NK cells. For example, CCL11, which was upregulated in both intercaruncular and caruncular tissue in pregnant animals attracts CCR3-expressing TH2 cells [31], and CCL2, which was only upregulated in caruncular tissue, attracts leukocytes expressing its receptor CCR2 [31,32]. Consistent with this is the association of CCL2 upregulation with immune tolerance in endometriosis, a mechanism suggested to act through its action on the FAS ligand, inducing apoptosis of T lymphocytes [33]. The FAS ligand, along with FAS and the downstream effector molecules FADD and caspase were all upregulated in pregnant animals in both tissue types. Another NK cell attracting chemokine up-regulated in pregnant animals was CCL8 [34]. This chemokines was upregulated 17 fold in the caruncular endometrium, and 9 fold in the intercaruncular endometrium. In addition to attracting NK cells, CCL8 can attract monocytes, lymphocytes, eosinophils, and basophils through its capacity to bind to the CCL2 receptor CCR2 as well as CCR1, CCR3 and CCR5. Both CCR1 and CCR5 were upregulated in caruncular and intercaruncular tissues of pregnant animals. Co-expression of proteases that can convert CCL8 to CCL8(6-75) results in an anti-inflammatory response, as CCL8(6-75) can inhibit other chemokines through its ability to act as a receptor antagonist [35].

Several interleukins that were up-regulated in pregnant animals may function to increase the presence of immune tolerance promoting T-regulatory (T-reg) cells in the uterus, as well as shifting the inflammatory balance towards an anti-inflammatory response. T-reg cells require low levels of some cytokines in order to differentiate from naive CD4+ T cell precursors, with high levels blocking suppression. In particular, IL-15, which was up-regulated 2 fold in the caruncular endometrium of pregnant animals. IL-15 also induces proliferation of T-reg cells [36]. Another cytokine that was up-regulated in the pregnant endometrium, IL-7, is considered a growth and survival promoting factor for T-reg cells [37]. Interleukin1β (IL-1β) and interleukin 18 (IL-18) are pro-inflammatory cytokines that were up-regulated in pregnant animals. Caspase 1, which proteolytically cleaves the IL-1β precursor to its active form, was also up-regulated in the caruncular endometrium of pregnant animals. Inhibitors of these cytokines, IL-1RN and IL-18BP were also up-regulated in pregnant animals. IL-1RN has recently been reported to be up-regulated in the pregnant equine endometrium [38], and down-regulated during the window of implantation in the cyclic human [39].

The TGFβ superfamily is a large functionally diverse protein family which includes several subfamilies including activins, bone morphogenic proteins (BMPs) and growth differentiation factors (GDF) [40]. Members of this family were differentially regulated with respect to pregnancy in the current study including TGFβ1, 2 and 3, which were all down-regulated in pregnant animals. The TGF-β superfamily members have immunomodulatory/inflammatory actions, some of which may be important during pregnancy recognition and maintenance. However, in the current study TGFβ1, 2, and 3, TGFβ1I1, and BMP6 were all down-regulated in pregnant animals, while TGFβI was up-regulated in pregnant animals in caruncular endometrium. Other members of this family that were down-regulated in pregnancy include; myostatin, Inhibin β A and Inhibin β B, this is coupled with increased expression of follistatin (2.8 fold), an inhibitor of both activins and myostatin. While activins have immunomodulatory actions [41] the GDF, myostatin has been proposed to have a role in glucose metabolism [42] in the placenta [43], and in an endometrial cell line [44]. Myostatin may be an important regulator of glucose availability to the embryo through regulation of glucose in endometrial histotroph secretions, and later for fetal development through regulation of placental glucose transport. Reduced myostatin accompanied by increased follistatin indicates that this pathway is under tight control during early pregnancy.

#### Antigen presentation and complement related genes

Complement pathway genes are some of the most commonly up-regulated genes in this and previous studies of endometrial gene expression during the window of implantation [8,45-47]. In particular, several molecules involved in activation of complement via the classical pathway were up-regulated in pregnant animals (Figure 3). Upregulation of complement may serve to provide the developing embryo with protection against pathogens and/or immune complexes and apoptotic cells, an important adaptation given the local immune suppressive state necessary for successful embryo implantation and placentation. Molecules that protect against complement mediated cells are expressed in the endometrium and trophoblast [48]. Consistent with this observation, pentraxin 3 (PTX3) was up-regulated in pregnant animals, particularly in the caruncules. PTX3 binding to the C1q complex can activate or inhibit the classical complement pathway depending on the nature of the interaction [49,50]. Upregulation of PTX3 may function to control the complement cascade in the pregnant animal and prevent excessive inflammatory reactions. Another gene that was up-regulated and may function to inhibit complement mediated immune responses is SERPING1. SERPING1 is a progesterone-induced, immunosuppressive and anti-proliferative glycoprotein contained in uterine secretions [51]. SERPING1 can inhibit complement-mediated immune responses through inactivation of C1s. Upregulation of SERPING1 may function to suppress a complement-mediated immune response to fetal antigens, and may also be important for embryonic growth. SERPING1 has been proposed to support conceptus growth through its ability to sequester the pluripotent growth factor activin A. Because of its immunosuppressive and anti-proliferative properties, it has been hypothesized to prevent fetal allograph rejection through inhibition of lymphocyte proliferation in the uterus [51]. Other SERPIN's that were differentially regulated include; SERPINA9, SERPINA1 and SERPINB11.

Other genes that were up-regulated in pregnant tissue encode immunoproteasome subunits PSMB8 and PSMB9, and antigenic peptide transporters TAP1 and TAP2 [52]. These results identify modulation of the extent and specificity of antigen presentation as a mechanism likely required for pregnancy success.

MHC class 1 and class 2 molecules along with B2 M were identified as being up-regulated in pregnant animals. Up-regulation of MHC class 1 and B2 M in response to pregnancy and interferons has been demonstrated [53,54]. Downregulation of classical MHC class 1 molecules in trophoblast tissue is a well characterized mechanism proposed to prevent immunological attack of the embryo [5]. The expression of MHC molecules in uterine tissue during pregnancy is poorly characterised. It has been proposed that upregulation of MHC class 1 molecules and B2 M may compensate for loss of mucin expression in the endometrial luminal epithelium[53]. Mucin-1 (MUC-1) forms part of the glycocalyx barrier that provides innate immune protection against bacterial infections; downregulation of MUC-1 is thought to be required for embryo attachment as the ectoderm tail presents a steric hindrance to attachment [52]. MUC-1 was down-regulated in pregnant animals in the study reported here, suggesting this mechanism may be active in the bovine endometrium also. Furthermore, the non-classical MHC class 1 molecule HLA-G was up-regulated in pregnant animals, perhaps to support an immunosuppressive action on the maternal immune system.

#### Antimicrobial response genes

Several antimicrobial genes are up-regulated in the endometrium of pregnant animals. LBP and BPI, encode proteins that bind bacterial endotoxin. Both were up-regulated in pregnant animals, 19 fold and 6 fold respectively, as was the antibacterial gene LYZ1 which was up-regulated 2.4 fold in pregnant animals. Increased expression of these genes in pregnant animals may confer innate immune protection against potential bacterial infection during a time of local immune suppression, as occurs during pregnancy [55].

#### Other immune response related genes

Indoleamine -2,3 dioxygenase (IDO) and tryptophanyl-tRNA synthetase (TTS/WARS) were up-regulated 5.7 fold and 2.4 fold respectively in endometrium of pregnant cows' in the current study. Upregulation of IDO has been reported in other species [56-58] and is likely to have an immunosuppressive function in the cow. Munn et al (1998) described a novel mechanism by which the maternal system prevents fetal rejection through increased IDO expression, specifically through the suppression of T-cell activity. Dendritic cell expression of IDO causes increased catabolism of the essential amino acid tryptophan, and increased concentration of the tryptophan metabolites 3-OH-Kynurenine and 3-OH-anthranilic acid. Tryptophan catabolism reduces the amount of tryptophan available in the microenvironment for protein synthesis, and can thus prevent expansion of T-cells. In addition, tryptophan metabolites have immunosuppressive properties such that they cause T-cell apoptosis. Cells expressing IDO are protected against tryptophan starvation, possibly through expression of TTS/WARS. TTS is the only known aminoacyl-tRNA synthetase induced by interferon gamma. The formation of a tryptophan-tRNA complex results in the generation of a reservoir of tryptophan that may function to protect IDO expressing cells from tryptophan depletion. Upregulation of these genes suggests this mechanism is active in the pregnant bovine endometrium and is likely an important contributor to pregnancy success in dairy cattle.

Several galectins were differentially expressed between pregnant and cyclic animals. Galectin 9 was up-regulated 2.8 fold in pregnant cows while galectin 8 and galectin 3 binding protein (caruncular only) were up-regulated 1.7 and 2.2 fold respectively. In contrast, galectin 12 was down-regulated in pregnant animals (1.6 fold) compared to cyclic animals. Galectins are a family of lectins that bind to beta-galactoside motifs in ligands such as laminin, fibronectin, and mucins in bivalent or multivalent ways, to modulate cellular adhesion [59]. Galectin 3 binding protein (Lgals3bp) can bind to Galectin -1, -3 and -7, promoting cell-cell adhesion through bridging between galectin molecules bound to ECM components [60,61]. It can also self-assemble to form high molecular weight complexes that promote cell adhesion through binding of integrins, collagens and fibronectin independently of Galectin 3 [61]. It has been demonstrated that Galectin-9 administration improves the survival of allogenic skin grafts in mice, possibly through induction of host cytotoxic CD8a+ T cell apoptosis [62]. Galectin-9 inhibits the secretion of TH1 and TH17 type cytokines, and promotes the synthesis of TH2 type cytokines in vitro [63]. Galectin-9 is potentially involved in modulation of the immune system during early pregnancy through its ability to induce apoptosis of immune cells, including activated CD4+ and CD8+ T cells through Ca2+ calpain caspase 1 pathway [64].

Some of the most highly expressed genes in the pregnant animals in this study were guanylate binding proteins (GBP), which belong to a family of GTPases that also includes Mx proteins (up-regulated in pregnant animals). GBPs regulate inhibition of proliferation, invasion of endothelial cells and cell survival. GBPs bind to guanine nucleotides, they contain two binding motifs. GBP-1 is up-regulated in the human endometrium during the window of implantation [65], and GBP1 and GBP-3,-4,-5 are up-regulated in the bovine endometrium at day 18 of pregnancy [10]. Consistent with this premise, GBP-1,-2,-3,-4 and GBP-6-7 were up-regulated in pregnant animals in the current study. GBPs are up-regulated in patients with inflammatory bowel disease (IBD) and also found to be associated with epithelial tight junctions, where downregulation of GBP through siRNA causes an increase in barrier permeability [66]. It has recently been suggested that the functional significance of GBP upregulation is to protect cells against pro-inflammatory cytokine induced apoptosis [66].

### Difference in caruncular and intercaruncular endometrial tissue

The majority of genes differentially expressed between pregnant and cyclic animals are in both caruncular and intercaruncular tissues. However, the magnitude of differential expression between pregnant and cyclic endometrium differs in the two tissues, and there are some genes that are only differentially expressed in one of the two tissue types. The caruncules of the endometrium are specialised projections that are the site of embryo attachment. Caruncules become highly vascularised, and are the major site for small molecule and gaseous exchange[4]. In comparison, intercaruncular tissue is highly glandular and responsible for early nourishment of the embryo through secretions of large molecules into the uterus [67-69]. The magnitude of some of these gene expression changes differs considerably between the two tissue types. RSAD2 is the most differentially expressed gene (23 fold up-regulated in pregnant animals) in the comparison of pregnant versus cyclic animals. In this comparison, RSAD2 is 3 fold more differentially expressed in caruncular than it is in intercaruncular tissue. This difference occurs in most of the genes discussed. The larger difference in gene expression seen in the caruncular tissue (indicating a greater response to pregnancy), may reflect the role of this tissue in implantation. The caruncules, being the site of embryo attachment may demand more extreme gene expression changes that promote tolerance to the embryo, allowing it to form a closer physical relationship without immunological attack.

## Conclusion

The maternal immune system is actively surveying the uterine environment during early pregnancy. The embryo modulates this response, inducing expression of molecules in the endometrium that function to suppress the immune response and/or promote tolerance to the embryo. The current study demonstrates this response with widespread upregulation of immune response pathways. During this period of immune suppression the endometrium would be expected to be susceptible to infections; the endometrium must, therefore, actively express specific molecules for defence against foreign pathogens. Upregulation of genes of the innate immune response including antimicrobial response genes support this hypothesis. This system requires intricate control through expression of protective inhibitors in the endometrium, and raises the question of whether the embryo expresses these same inhibitory molecules.

## Methods

### Animals

All Procedures were undertaken with the approval of the Ruakura Animal Ethics Committee (Hamilton, New Zealand). The estrus cycles of 22 lactating dairy cows were synchronized and 12 of these received embryo transfer on day 7 of the estrus cycle. Embryos were at the blastocyst stage of development and of grade 1 quality. Animals were slaughtered at day 17 of the reproductive cycle and endometrial tissues (both caruncular and intercaruncular) were sampled. There were 12 pregnant and 10 cyclic animals representing mixed New Zealand and North American ancestry Holstein Friesian dairy cows. Further details, including production data is provided in Meier et al 2009[70].

### RNA Extraction

Tissues were homogenized in Qiagen buffer RLT (QIAGEN GmbH, QIAGEN Strasse 1, 40724 Hilden, Germany) using Fastprep Lysing Matrix D tubes in a FastPrep instrument (MP Biomedicals, 29525 Fountain Pkwy, Solon, OH 44139).

Total RNA was extracted using a Qiagen RNeasy kit (Qiagen). RNA quantity was determined by spectrophotometry using a Nanodrop ND-1000 (Nanodrop Technologies, Wilmington, DE). RNA integrity was assessed with the Agilent 2100 Bioanalyzer with a RNA 6000 Nano LabChip kit (Agilent Technologies, Palo Alto, CA).

#### Microarray

One μg of RNA was amplified using the amino Allyl MessageAmp ™ aRNA Kit (Ambion, 2130 Woodward St, Austin TX, 78744) to generate amino allyl modified aRNA for use in microarray hybridization. The aRNA quantity was measured by spectrophotometry using a ND-1000 (Nanodrop Technologies, Wilmington, DE).

Five μg of aRNA was then vacuum dried and labeled with Cy3 and Cy5 NHS ester (Amersham Cy3 and Cy5 Mono-Reactive Dye Packs, GE Healthcare UK Ltd., Little Chalfont, Buckinghamshire). Labeled aRNA was then purified on column. Labeling efficiency was determined by spectrophotometry using the Nanodrop 1000.

825 ng of Cy3 and Cy5 labeled and fragmented aRNA were added to Agilent 44 k 60-mer oligonucleotide microarrays (G2514F), hybridized overnight (17 hours), washed and air dried according to the manufacturer's instructions (Agilent Gene Expression Hybridization Kit 60-mer oligo microarray protocol version 4.0). Arrays were scanned using the Agilent DNA microarray scanner.

### Hybridization design

A total of 44 microarrays were used in this study, one for each tissue type of the 22 animals. A reference sample was utilized, made from equal amounts of RNA from each endometrial sample analyzed (22 caruncular and 22 intercaruncular samples). This pooled sample was used as a 'reference' in each array hybridization. The reference sample was labeled with the Cy3 NHS ester dye, while each individual sample was labeled with the Cy5 NHS ester dye.

### Data analysis and statistics

Agilent feature extraction software version 7.1 was used to analyse the scanned Agilent microarray. The 44 scanned microarray image files were uploaded to the feature extraction software. Using a design file (015354), the feature extraction software locates features and converts the extracted data from each feature into a quantitative log ratio. The software removes pixel outliers, performs statistical tests on the non outlier pixels, subtracts background from features and flags any outlier features. The software was then used to perform a LOWESS (locally weighted linear regression analysis) dye normalisation and to calculate a p-value for each feature.

Data analysis was performed with Genespring GX 7.3.1. (Agilent, Palo Alto, CA, USA). Microarray data were imported into Genespring using Agilent's two-color 'Enhanced FE' import scenario which included 'Per Spot: Divide by control channel' and 'Per Chip: Normalize to 50th percentile' normalization steps.

Filters applied to the data to improve the quality of the normalized dataset included; firstly, filtering 'on flags' to ensure any probes that were not deemed 'present or marginal' (according to feature extraction spot quality guidelines) in at least 22 of the 44 arrays were omitted from analysis; secondly, probes that did not have a minimum threshold of 80 raw intensity units in at least 22 of 44 arrays were also omitted from analysis (15,833 probes passed this filter). The raw intensity cut off value of 80 was determined based on the base over proportional (C = a/b) calculation, which is generated by plotting the standard deviation of normalized values against the control values. The point at which the curve flattens out is where the data measurement becomes reliable or where C (control strength) = a/b (where a = base and b = the proportional coefficient).

Differentially expressed probes were identified using a T-Test, including a Benjamini-Hochberg false discovery rate multiple testing correction (MTC). For probes that were not annotated, full length transcripts were identified where possible by querying microarray probe sequences against the bovine genome (Btau3,1) using NCBI BLAST (http://blast.ncbi.nlm.nih.gov/Blast.cgi). Microarray data was submitted to NCBI gene expression omnibus (GSE19140).

#### cDNA Synthesis

One μg of an RNA sample was used for cDNA synthesis using the Invitrogen Superscript III Supermix kit (Invitrogen, Carlsbad, California, USA). Total RNA was transcribed according to the manufacturer's instructions using 27 μM of random pentadecamers. Briefly, RNA and random primers were mixed and denatured at 65°C for 5 minutes, followed by 1 minute on ice. Annealing buffer and Superscript/RNase was added to samples and incubated for 10 minutes at 25°C (primer annealing), followed by 50 minutes at 50°C and 5 minutes at 85°C to inactivate the enzyme. Reverse transcription controls were performed, whereby the above process was completed without the addition of superscript enzyme.

#### Quantitative Real Time PCR

Real time PCR using the Roche Lightcycler 480 was performed using the Roche real time PCR master mix (Lightcycler 480 Probes Master) in combination with Roche Universal Probe Library (UPL) assays (Roche diagnostics, Indianapolis, IN, USA). Assays were designed using Roche UPL design software. All assays were designed to span an intron-exon boundary.

The PCR reaction volume was 10 μL, consisting of 0.5 μM of each primer and 0.1 μM of probe. Standard cycling conditions were used [95°C for 10 minutes, (95°C for 10 seconds, 60°C for 30 seconds) × 50 cycles, 40°C for 40 seconds].

Each PCR experiment included a reaction in which template was replaced by water, and a reaction omitting reverse transcriptase as controls. Triplicate measurements were performed for all samples and standard curves. The percent coefficient of variation (%CV) for Cps was calculated for each sample. All samples for each gene were run on the same plate.

### Absolute quantification

The Roche Lightcycler 480 software was used to perform absolute quantification analysis of gene expression using the standard curve second derivative maximum analysis method, which is a non-linear regression line method. A six point standard curve was used with a starting concentration of 1 and final concentration of 1.6E-03.

### Relative quantification

The Roche Lightcycler 480 Software was used to perform quantification using the 'advanced relative quantification analysis' algorithm. Two endogenous control genes were used to normalize the data, taking the geometric mean of the normalized ratio of target gene to each reference gene [71]. A calibrator sample was then used as a control, whereby each calculated expression value was normalized to the calibrator sample.

The RT-PCR results were compared to those obtained using the microarray for five differentially expressed genes (OXTR, IDO, SPP1, OAS2, and CXCL11). Correlation coefficients were calculated for all genes to compare the calculated gene expression data from qRT-PCR and the microarray data. Genes for RT-PCR were selected based on two criteria. Biological significance and fold change.

#### Gene function and pathway identification

Differentially regulated genes were annotated with biological and molecular functions using the PANTHER (Protein ANalysis THrough Evolutionary Relationships) Classification System (http://www.pantherdb.org/) [72]. Ingenuity pathway analysis (IPA - Ingenuity^® ^Systems, http://www.ingenuity.com) was used for biological network generation and functional analysis.

Gene lists containing differentially expressed genes for each comparison (pregnant versus cyclic, caruncular pregnant versus caruncular cyclic, and intercaruncular pregnant versus intercaruncular cyclic, 1.5 fold differential expression, P-value ≤ 0.5, Benjamini-Hochberg FDR MTC) were used for analyses. The human homologue corresponding to the bovine gene representing each transcript identified as being differentially expressed was used.

For Ingenuity pathway analysis, the above gene list was uploaded and a core analysis was performed. The default background (ingenuity knowledge base) was used for all analyses. Each gene in the uploaded list was assessed for network eligibility (determined by the representation of the gene in the Ingenuity pathway knowledge base). Each eligible gene was then mapped using the data contained within the Ingenuity knowledge base. Networks were generated based on connectivity of each of the genes, and a score (p-value calculation) was assigned based on likelihood that these genes are part of a network and did not associate due to random chance. This score was then used to rank each of the generated networks. Molecules that are known to have direct and indirect associations were used for network generation.

IPA was also used to identify significant 'biological functions'. A Fischer's exact test was used to calculate the probability that the assigned biological function was not due to random chance.

Canonical pathways contained within the IPA database were used to identify pathways that were significantly enriched in the dataset. The ratio of genes associated with each canonical pathway to the total number of genes in that pathway was calculated, and a Fisher's exact test was used to test the probability that the association of these genes was significant.

## Authors' contributions

CGW was involved in experimental design, performed the experimental work and statistical analysis, and drafted the manuscript. SM designed the animal trial, carried out sample collection, acted as project manager and obtained funding for the project. MDL was involved in experimental design, critical analysis of the manuscript, and general supervision of the project. KL designed the microarrays and was involved in critical analysis of the manuscript. JRR was involved in critical analysis of the manuscript and general supervision of the project. MDM was involved in experimental design, critical analysis of the manuscript and general supervision of the project. All Authors read and approved the final manuscript.

## Supplementary Material

Additional file 1 Table S1: List of differentially expressed genes between pregnant and cyclic animals (≥ 1.5 fold change in expression; P-value < 0.05, Benjamini-Hochberg MTC). Click here for file

Additional file 2 **Table S2: Top canonical pathways identified using IPA.** *Interferon stimulated genes not previously identified in the bovine endometrium. Click here for file

Additional file 3 Table S3: Genes identified as relating to immunity and defence through PANTHER analysis Click here for file
